# Laparoscopic gastric dissociation using a two-port approach in minimally invasive esophagectomy

**DOI:** 10.1186/s12957-022-02843-4

**Published:** 2022-11-30

**Authors:** Huaguang Pan, Renquan Zhang, Ao Li, Hanlin Fang, Hao Zheng, Menglong Jiang, Wei Ge, Fan Zhou, Xiancheng Liu, Chuntong Yin

**Affiliations:** 1 Department of Thoracic Surgery, The First Affiliated Hospital of Anhui Medical University, Hefei, 230022 Anhui Province China; 2 Department of Thoracic Surgery, Lixin County People’s Hospital, Bozhou, Anhui Province China; 3 Department of Surgery, China Railway Fuyang Central Hospital, Fuyang, Anhui Province China; 4 Department of Surgery, Yuexi County Hospital, Anqing, Anhui Province China

**Keywords:** McKeown, Two-port laparoscopy, Five-port laparoscopy, Esophageal cancer

## Abstract

**Background:**

A new approach for laparoscopic gastric dissociation in minimally invasive esophagectomy (MIE) was attempted. This study aimed to evaluate the short-term outcomes, safety, and efficacy of two-port laparoscopy using the McKeown procedure.

**Methods:**

This retrospective study included 206 consecutive patients with esophageal cancer who underwent a modified two-port laparoscopic or the traditional five-port McKeown procedure at our institution from August 2019 to August 2021. Surgical outcomes of the two methods were compared.

**Results:**

Of the 206 patients, 106 (51.46%) underwent the modified two-port procedure, whereas 100 (48.54%) underwent the traditional five-port procedure. Subsequently, 182 propensity score-matched patients were compared. No significant differences were observed in laparoscopic operative time, blood loss during laparoscopic surgery, number of dissected lymph nodes, and pain score on postoperative day 1 between the two groups. The rate of complication and postoperative length of hospital stay did not differ significantly between the two groups. The total hospitalization cost also did not differ significantly between the two groups (*p* = 0.325). No postoperative deaths occurred in either group.

**Conclusions:**

Our findings demonstrate that laparoscopic gastric dissociation using the two-port approach in MIE is a safe and effective procedure, with short-term outcomes comparable to those of the traditional five-port procedure in patients with esophageal cancer. Larger studies with longer follow-up duration are warranted.

## Introduction

Esophageal cancer is a common gastrointestinal malignancy, with surgery as the primary treatment option. With the promotion of minimally invasive technology and concept of enhanced recovery after surgery (ERAS), minimally invasive esophagectomy (MIE) has become increasingly popular because of several advantages, such as reduced trauma, rapid recovery, few postoperative pulmonary complications, and improved esthetic appearance [1–4]. In MIE, the traditional five-port procedure is routinely used for laparoscopic gastric dissociation, which can be facilitated by the assistance and exposure provided by surgical assistants. However, there are several abdominal incisions, and various complications, such as operative scar formation, incision bleeding, hernia formation of the puncture hole, and incision infection, often develop [5–7].

With the continuous pursuit of extremely minimally invasive techniques by surgeons, single- and reduced-port laparoscopic techniques have been developed in the fields of cholecystectomy and appendectomy as well as in gastric benign disease treatment and radical resection of gastric cancer, resulting in smaller abdominal incisions and improved esthetic appearance [8–10]. However, surgery for esophageal cancer is relatively more complex and includes gastric dissociation, tubular stomach construction, and esophagogastric anastomosis. To the best of our knowledge, no study to date has evaluated its feasibility in MIE. Therefore, in this study, we retrospectively analyzed the clinical data of 206 consecutive patients with esophageal cancer who were treated with MIE by the same operator to evaluate the short-term outcomes, safety, and feasibility of laparoscopic gastric dissociation using the two-port approach in MIE (i.e., modified two-port McKeown procedure) compared with the traditional five-port procedure. We also preliminarily summarized the technical experience.

## Patients and methods

### Patients

The inclusion criteria were as follows: (1) esophageal squamous cell carcinoma confirmed via preoperative endoscopic histopathological examination, (2) modified two-port laparoscopic or traditional five-port McKeown procedure on the abdomen, (3) preoperative clinical staging of esophageal cancer of cT_1-3_N_0-3_M_0_ (8th edition of the American Joint Committee for Esophageal Cancer Staging Manual), (4) American Society of Anesthesiologists rating of I–III, (5) ability to tolerate surgery based on the preoperative cardiopulmonary function examination, and (6) availability of complete clinical data.

The exclusion criteria were as follows: (1) history of upper abdominal surgery (except cholecystectomy), (2) complication with other malignant tumors, (3) body mass index (BMI) of ≥ 30 kg/m^2^, and (4) severe underlying disease.

According to the abovementioned criteria, 206 consecutive patients with esophageal cancer underwent the modified two-port laparoscopic or traditional five-port McKeown surgery at the First Affiliated Hospital of Anhui Medical University from August 2019 to August 2021. Patient data on baseline characteristics and outcomes were retrospectively collected. The surgical procedure was based on patient’s preference in all cases. All surgeries were performed by the same medical team, comprising surgeons experienced in performing more than 800 MIEs. This study was reviewed and approved by the Ethics Committee of the First Affiliated Hospital of Anhui Medical University (Quick-PJ 2022–06-16).

### Surgical procedure

**Fig. 1:**
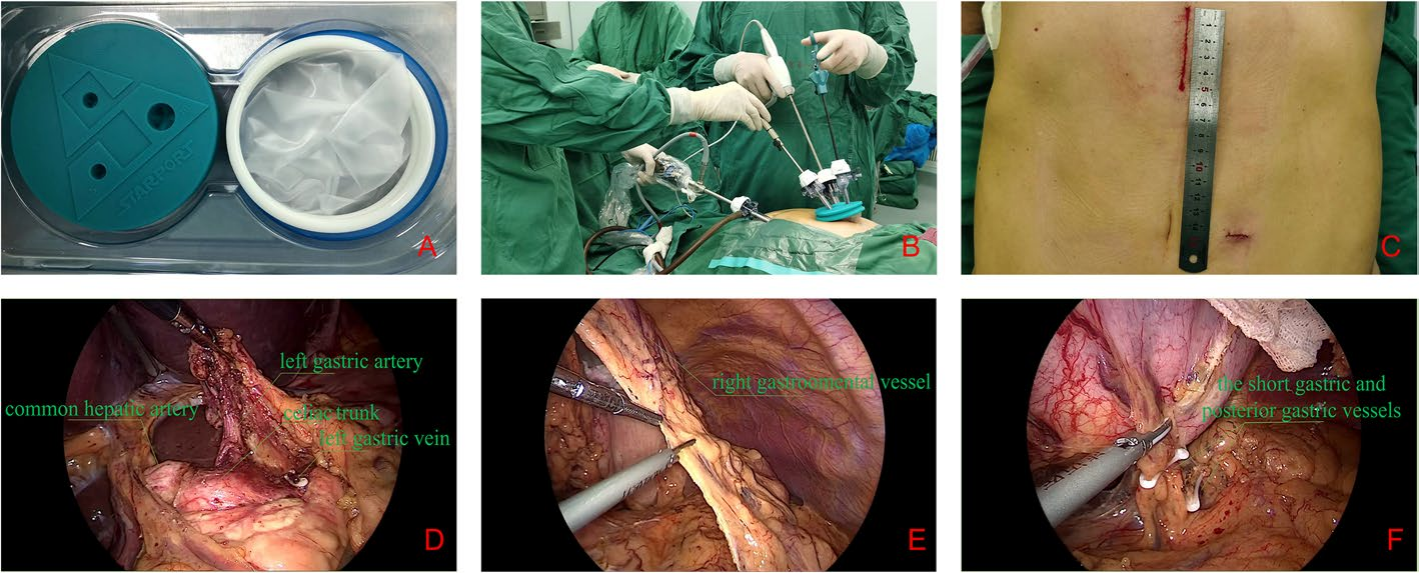
Modified McKeown procedure with two-port laparoscopy for esophageal cancer. **A** Disposable multichannel single-hole laparoscopic trocar (IA-3A-70 × 150; Schneider Xiamen Medical Instrument Co., Ltd.). **B** Abdominal operative position. **C** Abdominal incision. **D**–**F** Anatomical exposure of abdominal vessels

Preparation and establishment of the operating platform: Single-lumen endotracheal intubation anesthesia (left lateral position first) with artificial pneumothorax (pressure, 3 mmHg; flow, 3 L/min) was established. Four-port thoracoscopy was used for thoracic esophageal and mediastinal lymph node dissection. After the thoracoscopic phase, the patient was placed in the supine position with the head high and feet low by 30° and was tilted 20° to the right side so that the greater omentum and intestine are moved to the right lower abdomen, exposing the operative field of the abdominal cavity. In the modified McKeown group, the surgeon and first assistant stood on the right and left side of the patient, respectively. A 5-cm incision was made along the anterior edge of the left neck sternocleidomastoid muscle, and the esophagus was dissociated and cut off. An appropriately-sized circular stapler anvil was placed at the proximal end, and the distal end was sutured and connected to a sterilized gastric tube for traction. A small 4-cm incision was made in the middle of the upper abdomen 2 cm below the xiphoid process (indicating laparoscopic operative start time). After penetrating the abdominal cavity layer by layer, a disposable multichannel single-hole laparoscopic trocar (IA-3A-70 × 150; Schneider Xiamen Medical Instrument Co., Ltd.) was placed. Three puncture channels (12 mm × 1, 5 mm × 2) were arranged on the airtight cover of the multichannel single-hole puncture device. First, a 12-mm trocar was placed on the airtight cover to establish a pneumoperitoneum, and intra-abdominal pressure was maintained at 15 mmHg. After the establishment of a satisfactory pneumoperitoneum, the abdominal cavity was assessed for metastasis and adhesion via endoscopy, and a 12-mm trocar was placed 3 cm to the left of the umbilicus as an endoscopic observation hole. Subsequently, two 5-mm trocars were placed on the airtight cover, and the operating platform was established (Fig. 1A–C).

Laparoscopic surgical procedures: Conventional laparoscopic instruments were used, and the assistant used intestinal forceps to block the liver to the cephalic side for exposing the lesser omental sac. The surgeon separated the lesser omental sac to the right side of the cardia using an ultrasonic knife. Lymph nodes in groups 17, 18, and 20 were dissected along the common hepatic artery, left gastric artery, and celiac trunk in the posterior pancreatic space (the American Joint Committee on Cancer/Union for International Cancer Control esophageal cancer staging system, 8th edition) [11]. The left gastric artery and vein were dissected using an ultrasonic knife after double ligation with Hem-o-lok ligature clips at the root. The right gastro-omental artery was located, and the gastrocolonal ligament was separated using an ultrasonic knife. The free greater omentum was located in the direction of spleen and approximately 1 cm away from the right gastro-omental artery. A wet gauze with intestinal forceps was used to lift the fundus of the stomach upward to fully expose the short gastric and posterior gastric vessels, and a vascular clamp was used with an ultrasonic knife to disconnect the vessels. The fibrous connective tissue around the cardia and lower esophagus were separated using an ultrasonic knife so that the abdominal and chest cavities were completely connected at the diaphragmatic hiatus. The esophagus was dragged from the diaphragmatic hiatus to the abdominal cavity under laparoscopic vision. Gas flow into the abdominal cavity was stopped, following which the puncture outfit vent was opened, abdominal residual gas was slowly released, and airtight cover was removed (indicating laparoscopic operative end time). Through the small incision in the epigastric abdomen, the esophagus and stomach were raised extraperitoneally, and a tubular stomach was constructed. Subsequently, the tubular stomach was lifted from the mediastinal esophageal bed to the neck and anastomosed with the proximal esophagus. The airtight cover was used again to establish a pneumoperitoneum. The abdominal cavity was assessed for active bleeding using laparoscopy, and the surgery was ended (Fig. 1D–F).

For five-port laparoscopy, a similar preparation maneuver was used. The puncture point was made at 1 cm above the umbilicus, and an artificial pneumoperitoneum was established. For laparoscopic surgery, the incisions of the five ports were made as follows. A 12-mm incision was made exteriorly at 1 cm above the umbilicus and used as the laparoscopic port; further, 12-mm and 5-mm incisions were made at 3 cm away from the paraumbilical region and used as the main operative ports. Next, a 5-mm incision below the right costal margin and another 5-mm incision under the xiphoid process were made and used as the assisting ports. Mobilization of the stomach and abdominal lymphadenectomy were performed using traditional laparoscopic methods. Subsequently, a 4-cm subxiphoid vertical incision was made based on the original auxiliary port, through which the stomach was pulled out. Finally, a tubular stomach was constructed, and esophagogastric anastomosis was performed proximal to the esophagus.

### Postoperative care

After surgery, intravenous nutritional support was provided based on body weight. Following the resumption of defecation, the patient’s family or nursing workers was instructed to provide liquid diet through the indwelling nasointestinal tube and to appropriately reduce the amount of intravenous fluid. Chest radiography and routine blood examination results were reviewed on postoperative days 1 and 2. In the absence of any complications, defined as a drainage volume of < 200 mL for 3 consecutive days and no air leakage, the drainage tube was removed on postoperative day 4 and the patient was discharged with a nasointestinal tube on the following day. Upper gastrointestinal radiography using water-soluble iodine-based contrast agent was performed approximately 2 weeks after the surgery, and the nasointestinal tube was removed after confirming the absence of contrast-enhanced external fistula. The patient was then instructed to resume oral feeding.

### Study outcomes

The primary study endpoint was the number of dissected celiac lymph nodes. The operative time was defined as the time from making the abdominal skin incision to the removal of the airtight cover. Morbidities were defined as complications requiring extended hospital stay or readmission. Postoperative complications included those occurring during the initial 30 days after surgery.

### Statistical analysis

To minimize the effect of basic clinical data on the outcomes of the two sets, we used one-to-one propensity score matching (PSM) analysis, and the absolute normalized mean difference of the variables after matching using a caliper of 0.05 can be considered to indicate matching equilibrium. Variables included age, sex, BMI, and tumor location. The SPSS 22.0 (IBM, Armonk, New York) software was used for PSM and statistical analyses. Continuous and categorical data were compared using the two-tailed *t*-test and *χ*^2^ test, respectively. A *p-*value of < 0.05 was considered statistically significant.

## Results

A total of 206 patients successfully underwent the modified two-port or traditional five-port McKeown procedure for esophageal cancer between August 2019 and August 2021 in our institution. The clinicopathologic features of the patients in both groups are presented in Table 1. Using PSM, 182 patients were included for comparison. Owing to extensive abdominal adhesion, two cases in the traditional five-port group were converted to open surgery, whereas in the modified two-port group, only one case added with a 12-mm auxiliary port in the right paraumbilical region.

**Table 1:** Patient demographic characteristics

Variables	All patients			Propensity-matched patients		
	Modified two-port McKeown procedure (*n* = 106)	Traditional five-port McKeown procedure (*n* = 100)	*p-*value	Modified two-port McKeown procedure (*n* = 91)	Traditional five-port McKeown procedure (*n* = 91)	*p-*value
Age (years)	67.76 ± 8.84	66.38 ± 7.58	0.230	66.66 ± 8.42	66.90 ± 7.55	0.839
Sex			0.191			1.000
Male	92 (86.8%)	80 (80.0%)		77 (84.6%)	77 (84.6%)	
Female	14 (13.2%)	20 (20.0%)		14 (15.4%)	14 (15.4%)	
BMI (kg/m^2^)	21.96 ± 2.80	22.21 ± 2.70	0.508	21.90 ± 2.86	22.13 ± 2.77	0.580
Smoking	23 (21.7%)	19 (19.0%)	0.633	18 (19.8%)	17 (18.7%)	0.852
ASA grade			0.742			0.489
I–II	85 (80.2%)	80 (80.0%)		79 (86.8%)	80 (87.9%)	
III	21 (19.8%)	20 (20.0%)		12 (13.2%)	11 (12.1%)	
Comorbidity						
Hypertension	13 (12.3%)	15 (15.0%)	0.443	11 (12.1%)	11 (12.1%)	1.000
Diabetes	8 (7.5%)	6 (6.0%)	0.881	7 (7.7%)	6 (6.6%)	0.775
Chronic lung disease	5 (4.7%)	7 (7.0%)	0.487	5 (5.5%)	6 (6.6%)	0.757
Arrhythmia	6 (5.7%)	5 (5.0%)	0.834	5 (5.5%)	5 (5.5%)	1.000
Other comorbidities	5 (4.7%)	2 (2.0%)	0.279	4 (4.4%)	2 (2.2%)	0.409
Tumor location			0.466			0.525
Upper	2 (1.9%)	10 (10.0%)		2 (2.2%)	8 (8.8%)	
Middle	65 (61.3%)	51 (51.0%)		53 (58.2%)	46 (50.5%)	
Lower	39 (36.8%)	39 (39.0%)		36 (39.6%)	37 (40.7%)	
Preoperative therapy			0.114			0.098
ESD	5 (4.7%)	1 (1.0%)		5 (5.5%)	1 (1.1%)	
Neoadjuvant chemotherapy	2 (1.9%)	0 (0.0%)		2 (2.2%)	0 (0.0%)	

Values are expressed as mean ± standard deviation or *n* (%)

*BMI*, body mass index; *ASA*, American Society of Anesthesiologists; *ESD*, endoscopic submucosal dissection; *PC*, postoperative complications

As shown in Table 2, the total operative time was 230.44 ± 46.31 and 237.74 ± 45.46 min in the modified two-port and traditional five-port groups, respectively (*p* = 0.285). Meanwhile, the laparoscopic operative time was 48.40 ± 13.33 and 45.75 ± 10.65 min, respectively (*p* = 0.140). Blood loss during laparoscopic surgery in the modified two-port group was comparable to that in the traditional five-port group (16.87 ± 18.93 vs. 15.88 ± 17.62 mL, *p* = 0.716). There was no significant difference in the median number of dissected celiac lymph nodes between the modified two-port and traditional five-port groups (7.69 ± 3.37 vs. 8.56 ± 6.54; *p* = 0.262), and the number of positive celiac lymph nodes was 0.36 ± 1.01 and 0.64 ± 1.32, respectively (*p* = 0.116). Although the histologic tumor type differed significantly between the two groups, it was not associated with the number of dissected celiac lymph nodes (*p* = 0.015).

**Table 2:** Perioperative clinical data of patients with esophageal cancer

Outcomes	Propensity-matched patients		
	Modified two-port McKeown procedure (*n* = 91)	Traditional five-port McKeown procedure (*n* = 91)	*p-*value
Total operative time (min)	230.44 ± 46.31	237.74 ± 45.46	0.285
Laparoscopic operative time (min)	48.40 ± 13.33	45.75 ± 10.65	0.140
Blood loss during laparoscopic surgery (mL)	16.87 ± 18.93	15.88 ± 17.62	0.716
Number of dissected celiac lymph nodes	7.69 ± 3.37	8.56 ± 6.54	0.262
Number of positive celiac lymph nodes	0.36 ± 1.01	0.64 ± 1.32	0.116
Histology			0.015
Squamous cell carcinoma	88 (96.7%)	79 (86.8%)	
Adenocarcinoma or others	3 (3.3%)	12 (13.2%)	
T stage			0.398
T1	29 (31.9%)	22 (24.2%)	
T2	13 (14.3%)	17 (18.7%)	
T3	49 (53.8%)	52 (57.1%)	
N stage			0.737
N0	52 (57.1%)	52 (57.1%)	
N1	24 (26.4%)	23 (25.3%)	
N2	12 (13.2%)	10 (11.0%)	
N3	3 (3.3%)	6 (6.6%)	
Nerve invasion	45 (49.5%)	42 (46.2%)	0.658
Vascular invasion	38 (41.8%)	31 (34.1%)	0.287
PC			0.563
Pulmonary inflammation	8 (8.8%)	8 (8.8%)	
Anastomotic leakage	3 (3.3%)	7 (7.7%)	
Persistent air leakage	1 (1.1%)	2 (2.2%)	
Postoperative hospital stay (days)	10.21 ± 4.23	10.22 ± 4.24	0.986
Hospitalization cost (yuan)	58,371.2 ± 3213.6	57,896.5 ± 4234.3	0.325

Values are expressed as mean ± standard deviation or *n* (%)

*PC*, postoperative complication

Complication rates of the patients in the modified two-port and traditional five-port groups did not differ significantly (13.2% vs. 18.7%, *p* = 0.563). The most common complication in the modified two-port group was pulmonary inflammation (8 patients), followed by anastomotic leakage (3 patients). A similar result was noted in the traditional five-port group. The postoperative length of hospital stay was 10.21 ± 4.23 and 10.22 ± 4.24 days for the modified two-port and traditional five-port groups, respectively (*p* = 0.986). More importantly, the total hospitalization cost did not differ significantly between the modified two-port and traditional five-port groups (58,371.2 ± 3213.6 vs. 57,896.5 ± 4234.3 yuan, *p* = 0.325). The pain scores on postoperative days 1, 3, and 5 were comparable between the two groups (*p* = 0.685, 0.366, and 0.786, respectively) (Table 3). No patients developed postoperative incisional hernias, and no perioperative deaths occurred in either group.

**Table 3:** Repeated-measures analysis of variance of postoperative abdominal pain VAS scores

Group	Propensity-matched patients		
	Modified two-port McKeown procedure (*n* = 91)	Traditional five-port McKeown procedure (*n* = 91)	*p-*value
POD #1	4.1 ± 0.9	4.0 ± 0.8	0.685
POD #3	3.2 ± 0.9	3.3 ± 0.8	0.366
POD #5	2.7 ± 0.8	2.8 ± 0.7	0.786

*VAS*, visual analog scale; *POD*, postoperative day

## Discussion

With the continuous promotion and popularization of thoracoscopy, thoracic surgeons continue to make breakthroughs based on previous surgical techniques, and incisions are becoming smaller. In particular, the previous four-port surgery in lung resection has gradually transitioned to three-, two-, and single-port surgery, which is currently widely performed [12]. A multicenter open-label randomized controlled trial reported a lower incidence of postoperative short-term pulmonary infections, shorter hospital stay, and better short-term quality of life in patients undergoing MIE than in those undergoing open esophagectomy [13]. Breakthroughs in MIE to further reduce operative scar formation and surgical trauma under the premise of radical treatment of esophageal tumors are needed in thoracic surgery.

Recently, an increasing number of general surgeons have successfully completed cholecystectomy, appendectomy, radical resection of colon cancer, and even radical resection of gastric cancer with a 3–4-cm incision at the lower umbilical margin, thus resulting in reduced trauma and pain, rapid recovery, and improved esthetic appearance [14, 15]. However, because the laparoscope and operator’s instruments entering the abdominal cavity through a single-hole puncture device, the operator’s manipulation may cause the endoscope to shake, resulting in unstable screen display; therefore, the laparoscopic assistant should firmly support the endoscope with both hands, keep the lens stable, flexibly adjust the lens angle, avoid the operator’s instruments, prevent collisions, keep the operator’s instruments located in front of the endoscope, avoid frequent lens swing, and minimize the operator’s dizziness and eye fatigue. The laparoscopic assistant should be familiar with the surgical steps and have long-term experience in cooperating with the surgeon. To avoid obstruction of the visual field by the liver, purse suture is often used to lift the liver lobes and fix them in vitro, thus providing good exposure for dissociating the stomach and dissecting abdominal lymph nodes.

In common MIE abdominal procedures, a 4-cm incision is made in the middle epigastrium of the subxiphoid to remove the esophageal tumor, construct a tubular stomach, and guide the placement of the nasointestinal tube. Therefore, in this study, considering the relevant experience of the general surgeon and characteristics of esophageal surgery in the thoracic cavity, we attempted to take maximum advantage of the small incision and made all operation ports, except the endoscopic observation ports, through the small incision. Previously, the endoscopic observation port was usually placed at the lower margin of the umbilicus; however, in the modified “two-port method,” the observation port was selected at 3 cm to the left of the umbilicus, which could avoid interference from the endoscope and operating instrument; simultaneously, the ultrasonic knife could be inserted into the observation port to dissect the short gastric vessels more conveniently. Moreover, if necessary, a latex drainage tube can be placed using the observation port. Ultimately, PSM to compare the short-term outcomes of the 182 patients revealed no significant differences in the laparoscopic operative time, blood loss during laparoscopic surgery, number of dissected lymph nodes, and pain score on postoperative day 1 between the two groups. The complication rate, postoperative length of hospital stay, and, more importantly, the total hospitalization cost did not differ significantly between the two groups. Moreover, no postoperative deaths occurred in either group.

Some advantages of this approach should not be overlooked. During abdominal surgery, we first established a small incision in the epigastric abdomen, which can determine the presence of adhesion in the abdominal cavity under direct vision; subsequently, a disposable multichannel single-port laparoscopic puncture device was placed to establish the pneumoperitoneum, thereby avoiding the risk of intestinal injury and bleeding caused by a pneumatic needle or direct penetration of the puncture. Simultaneously, the operation can be immediately converted to open surgery. Although the position of the three puncture ports on the airtight cover is relatively fixed, we can rotate the airtight cover according to the needs of the operation and adjust the relative position of each puncture port to facilitate the operation. Additionally, the instrument and lens can be freely moved between the two ports depending on the surgical area and operative requirements, thus reducing the difficulty associated with the surgery. During the surgery, we selected a disposable multichannel single-hole laparoscopic puncture device with a diameter of 7 cm, which was slightly larger than the abdominal incision of 4 cm, causing difficulty in removing the puncture device through the surgical incision. This led to the establishment of a more reliable pneumoperitoneum and expansion of the operating space for surgery. After completing the laparoscopic surgery, the airtight cover was removed and the esophageal tumor and stomach were dragged out under the protection and along the extension of the incision via a laparoscopic puncture instrument, thereby avoiding the possibility of abdominal incision implantation of the tumor. This makes it easier to release the adhesion connective tissue around the pylorus and place the prepared tubular stomach into the abdominal cavity. When the surgery was completed, the airtight cover was applied again to rapidly establish a pneumoperitoneum, and the abdominal cavity was examined for active bleeding via laparoscopy.

The modified two-port McKeown procedure for esophageal cancer may exhibit a certain degree of difficulty during the initial application. We optimized the surgical ideas and methods according to our own experience in the following manner: (1) the previously used operation sequence involved the abdomen followed by the neck; we adjusted this sequence by disconnecting the esophagus in the neck and then performing abdominal surgery. In this way, the lower esophagus and cardia can be fully dissociated after abdominal dissociation of the stomach as well as when the short gastric or posterior gastric vessels are obstructed. Thus, dragging the lower esophagus into the abdominal cavity and then treating the blood vessels from the rear side can greatly reduce the difficulty associated with surgery and risk of bleeding. (2) Instead of the entire palm, the right middle and index fingers were used to guide the nasointestinal tube through the small abdominal incision.

The following problems should also be noted: first, patients should be carefully selected in the early stage of the technology. It is recommended to select patients with no history of abdominal surgery, slim build, and long epigastric length to reduce the difficulty and risk of surgery. Second, in case of difficulties, laparotomy should be performed as soon as possible or an operative port should be added. The quality and time of surgery should not be neglected to complete the procedure. During our procedure, a 12-mm auxiliary port was added to the right side of the umbilicus in one patient because of extensive adhesion in the abdominal cavity after previous open cholecystectomy. For patients with obesity or severe adhesions around the pylorus, a small incision in the abdomen can be appropriately extended to 5 cm to achieve direct vision for separating the remaining parts, which is convenient for exposure, preventing injury to the right gastro-omental artery, and adequately releasing the adhesions.

However, our study has some limitations. First, this was a retrospective study involving only one surgeon, and no randomized approach was used for the selection of patients in both groups. In addition, according to the Clavien–Dindo complication grading system, both groups were classified as grade I and could not be studied further [16]. Therefore, the study findings cannot be generalized to large populations or other centers. Second, although we utilized PSM to minimize the effects of confounding factors, the predominant histologic tumor type was squamous cell carcinoma. Finally, owing to the relatively short study duration, complete data on long-term survival and recurrence were not available. Therefore, further prospective and multicenter clinical studies are warranted to clarify these aspects.

In summary, this study revealed that the modified “two-port method” in MIE exhibits good operability and safety in lymph node dissection and gastric dissociation, and surgical trauma is reduced following the principle of tumor-free operation and standard lymph node dissection. Currently, single-port and reduced-port laparoscopy is the most popular minimally invasive technology, which not only indicates the origin and innovation of the traditional five-port laparoscopic technology but also represents the direction of the development of precision minimally invasive technology. Moreover, single-port and reduced-port laparoscopic techniques meet the development needs of the contemporary concept of ERAS. Considering the lower invasiveness and better cosmetic outcomes of the modified two-port method, this approach is expected to be the next step in reduced-port laparoscopy.

## Data Availability

The data underlying this article will be shared on reasonable request to the corresponding author.
